# RNA-Seq Data for Reliable SNP Detection and Genotype Calling: Interest for Coding Variant Characterization and *Cis*-Regulation Analysis by Allele-Specific Expression in Livestock Species

**DOI:** 10.3389/fgene.2021.655707

**Published:** 2021-06-28

**Authors:** Frédéric Jehl, Fabien Degalez, Maria Bernard, Frédéric Lecerf, Laetitia Lagoutte, Colette Désert, Manon Coulée, Olivier Bouchez, Sophie Leroux, Behnam Abasht, Michèle Tixier-Boichard, Bertrand Bed’hom, Thierry Burlot, David Gourichon, Philippe Bardou, Hervé Acloque, Sylvain Foissac, Sarah Djebali, Elisabetta Giuffra, Tatiana Zerjal, Frédérique Pitel, Christophe Klopp, Sandrine Lagarrigue

**Affiliations:** ^1^INRAE, INSTITUT AGRO, PEGASE UMR 1348, Saint-Gilles, France; ^2^INRAE, SIGENAE, Genotoul Bioinfo MIAT, Castanet-Tolosan, France; ^3^INRAE, AgroParisTech, Université Paris-Saclay, GABI UMR 1313, Jouy-en-Josas, France; ^4^INRAE, US 1426, GeT-PlaGe, Genotoul, Castanet-Tolosan, France; ^5^INRAE, INPT, ENVT, Université de Toulouse, GenPhySE UMR 1388, Castanet-Tolosan, France; ^6^Department of Animal and Food Sciences, University of Delaware, Newark, DE, United States; ^7^NOVOGEN, Maugueérand, Le Foeil, France; ^8^INRAE, PEAT UE, Nouzilly, France

**Keywords:** RNA-seq, SNP calling, genotype calling, SNP annotation, allele-specific expression, livestock, chicken

## Abstract

In addition to their common usages to study gene expression, RNA-seq data accumulated over the last 10 years are a yet-unexploited resource of SNPs in numerous individuals from different populations. SNP detection by RNA-seq is particularly interesting for livestock species since whole genome sequencing is expensive and exome sequencing tools are unavailable. These SNPs detected in expressed regions can be used to characterize variants affecting protein functions, and to study *cis*-regulated genes by analyzing allele-specific expression (ASE) in the tissue of interest. However, gene expression can be highly variable, and filters for SNP detection using the popular GATK toolkit are not yet standardized, making SNP detection and genotype calling by RNA-seq a challenging endeavor. We compared SNP calling results using GATK suggested filters, on two chicken populations for which both RNA-seq and DNA-seq data were available for the same samples of the same tissue. We showed, in expressed regions, a RNA-seq precision of 91% (SNPs detected by RNA-seq and shared by DNA-seq) and we characterized the remaining 9% of SNPs. We then studied the genotype (GT) obtained by RNA-seq and the impact of two factors (GT call-rate and read number per GT) on the concordance of GT with DNA-seq; we proposed thresholds for them leading to a 95% concordance. Applying these thresholds to 767 multi-tissue RNA-seq of 382 birds of 11 chicken populations, we found 9.5 M SNPs in total, of which ∼550,000 SNPs per tissue and population with a reliable GT (call rate ≥ 50%) and among them, ∼340,000 with a MAF ≥ 10%. We showed that such RNA-seq data from one tissue can be used to (*i*) detect SNPs with a strong predicted impact on proteins, despite their scarcity in each population (16,307 SIFT deleterious missenses and 590 stop-gained), (*ii*) study, on a large scale, *cis*-regulations of gene expression, with ∼81% of protein-coding and 68% of long non-coding genes (TPM ≥ 1) that can be analyzed for ASE, and with ∼29% of them that were *cis*-regulated, and (*iii*) analyze population genetic using such SNPs located in expressed regions. This work shows that RNA-seq data can be used with good confidence to detect SNPs and associated GT within various populations and used them for different analyses as GTEx studies.

## Introduction

RNA-seq is currently the method of choice to study transcriptome expression in replacement of gene chips (Mortazavi et al., 2008). This technology is commonly used to study gene expression patterns in a variety of organisms including plant, animal or human groups to better understand the genetic mechanisms intervening in the determinism of phenotypes (Gondret et al., 2017), diseases (Savary et al., 2020) or response to environmental changes (Jehl et al., 2019) among others. The RNA-seq has other more specific applications taking advantage of its sequencing step. For example RNA-seq allows transcript and gene modeling as shown by long non-coding atlas reported in different species (Derrien et al., 2012; Jehl et al., 2020). It also allows to combine SNP information, at the RNA level with gene expression to study the variation which affects gene-expression levels: it is a powerful technology to identify such expression quantitative trait locus (eQTL) either through GWAS mapping (if the individual number is sufficient) or through allele-specific expression (ASE) analysis as shown by growing number of studies on a variety of species since the beginning of the RNA-seq technology in the 2010s (Montgomery et al., 2010; Pickrell et al., 2010; Battle et al., 2013; Lagarrigue et al., 2013b; Chamberlain et al., 2015; Deelen et al., 2015; The GTEx Consortium, 2020), among them the famous studies from the human GTEx consortium (The GTEx Consortium, 2020). Finally RNA-seq allows RNA editing analysis, a phenomenon resulting in nucleotide changes observed at RNA level, occurring after its transcription from DNA level (Kleinman et al., 2012). In these two last applications, RNA-seq is in general combined with DNA-seq used for genotyping individuals. However, RNA-seq can also detect genomic variations in expressed regions like DNA-seq, as described by Piskol et al. (2013). It is particularly interesting in non-model species (wild or domesticated, for example livestock species) in which no exome capturing tools have been developed as an alternative to DNA-seq data, which remains costly to generate and store. In this context, RNA-seq presents several advantages compared to the DNA-seq. First, the number of RNA-seq data sets publicly available is much higher than the number of DNA-seq data sets, for many species (chicken, pig, cow, and other non-model species) since these data have accumulated over the past several years and continue to accumulate in different populations and within populations. Moreover, within populations, different conditions are studied, increasing the number of studied animals, allowing to better detect, in a given population, variants with low frequencies. Second, RNA-seq data allows studying coding region variations that have potential functional impacts. Some of these SNPs can induce a loss of the protein function. These loss-of-function variants are extensively studied because of their possible contribution to phenotypes (Genome Aggregation Database Consortium et al., 2020). In addition, they represent a powerful source of information to understand gene functions (Genome Aggregation Database Consortium et al., 2020). However, these loss of function SNPs are rather rare because purged by negative selection in natural populations but can be detected with a certain number of samples. In well-known model-species or human, these coding region variants are accessible using whole exome sequencing (WES), as shown by the recent work of the Genome Aggregation Database (gnomAD) (Lek et al., 2016). This consortium analyzed 125,748 human exomes (and much fewer whole genomes: 15,708) from public sources and identified 443,769 high-confidence predicted loss-of-function variants, defined in the work of gnomAD as being either gain of stop (non-sense variants), frameshift or splice site variants. For non-model species such as livestock species, for which the WES method is usually not available, RNA-seq can thus fulfill the same objective, with a similar advantage that is, producing a smaller data volume, thus facilitating data storage and decreasing costs (Battle et al., 2013). Third, RNA-seq data provides expression levels of loci harboring SNPs, allowing to study allele-specific expression as we previously mentioned, and hence, to study *cis*-regulation on a large scale, in multiple tissues and multiple populations. Fourth, the transcribed regions are well spread over the genome and much more numerous than previously thought. Thousands of novel long non-coding genes exist across the genome, as highlighted by the ENCODE project (Derrien et al., 2012). RNA-seq data can therefore provide sets of numerous and well distributed SNPs throughout the genome. Finally, these data could be used to study population genetic diversity from a different point of view compared to the SNP chips, by offering various sets of SNPs with more or less severe functional impacts and not neutral SNPs.

**FIGURE 1:**
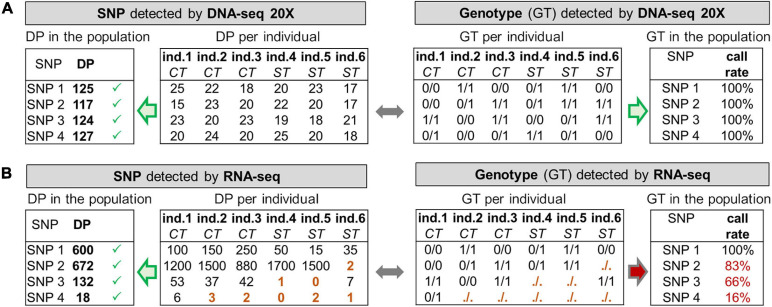
Toy example with simulated data illustrating the need for read depth (DP) filters in RNA-seq and differences with DNA-seq. **(A)** DNA-seq data offers a globally homogeneous genome coverage (20X in our case), all SNPs are therefore detected by GATK at the individual level with a DP of 20 reads on average (*“DP per individual”*), and at the population level with a DP of 6 × 20 = 120 reads on average (*“DP in the population* resulting from the addition of *”DP per individual”)*. All genotypes (GT) can therefore be computed at the individual level (*“GT per individual”*), resulting in a genotype call rate of 100% for every SNP (*“GT in the population”*). **(B)** RNA-seq data offers a heterogeneous coverage of the genome depending on the expression of the genes harboring the SNPs. *At the population level*, 4 SNPs having a sufficiently high DP are detected by GATK. At the individual level, SNP 1 shows good read coverage across all samples whereas SNP 3 is on a gene that has a lower expression, in particular in the stress (ST) condition compared to the control (CT). SNP 4 is on an overall very lowly expressed gene. *In terms of genotype (GT) per individual*, some cannot be provided by GATK (noted “./.”) because of a too low DP (i.e., 5 reads, see brown GT and DP) and are not considered for the GT call rate. For SNP 3, most of the individuals from the ST condition have no GT and for SNP 4, only one GT is called whereas in both case the SNP is detected at the population-level. “GT in the population” provides for each SNP their call-rate for the genotypes (CR): SNP 1 has 100% of the samples with a GT whereas SNP 4 has 16% and cannot be used to compute meaningful genotype frequencies.

Despite the aforementioned advantages RNA-seq is not yet often used for SNP detection in coding regions. Indeed, SNP detection and genotype calling by RNA-seq present three main challenges. First, the transcriptome is composed of mature transcripts (i.e., spliced), making mapping of RNA-seq reads that overlap exon-exon junctions, more difficult, compared to DNA-seq read alignment (Pan et al., 2008). However, RNA-seq mapping methods seem to be well mastered in recent years, even though it is important to remain cautious for SNPs detected close to exon-exon junctions (Peng et al., 2012; Lagarrigue et al., 2013b). Second, RNA editing, by definition, could represent a strong limitation for SNP detection by RNA-seq, mainly because it introduces variations at the RNA level, which are absent at the DNA level. Nevertheless, as we will discuss later, RNA editing has such features that it only slightly impedes reliable RNA-seq based variation detection in standard conditions. Third, genes exhibit highly variable expression levels, leading to the read depths ranging from a few reads to millions of reads, contrarily to the DNA-seq which offers a rather homogeneous read depth across the genome (see Figures 1A,B). Indeed, coding and non-coding transcripts can be expressed at vastly different levels, ranging from few copies to millions of copies per cell, in different cell types and developmental or physiological stages. Moreover, the transcriptome is also composed of a small portion of immature under processing transcripts (composed of exons and introns), less supported by reads but enriched in introns that are more variable in sequence compared to exons (Sims et al., 2014). In summary, these variations in read depth from one gene to another, and within a gene (between introns and exons) constitute a major challenge for SNP detection (see Figures 1A,B, left), and more importantly, for individual genotype calling (see Figures 1A,B, right). Indeed, reliable SNP detection at the population level benefits from the information accumulation born by the reads across individuals, in contrast to genotype calling. This last point might explain why only few studies have used RNA-seq data for variant detection and genotype calling since the first publications. Consequently, neither the number of SNPs that could be detected using RNA-seq, nor the percentage of individuals with a given genotype (a prerequisite for computing allelic frequencies), are known. To our best knowledge, since Piskol et al. (2013), less than a dozen studies were focused on large-scale SNP detection tools from RNA-seq data (Quinn et al., 2013; Tang et al., 2014; Wang et al., 2014; Wolfien et al., 2016; Oikkonen and Lise, 2017; Cornwell et al., 2018; Adetunji et al., 2019). The reference tools for read mapping and variant detection have been evolving very rapidly, and these studies have tested different tools, and among them, only Adetunji et al., 2019 (Adetunji et al., 2019) used the most recent tools proposed by ENCODE for RNA-seq data, i.e., STAR (Dobin et al., 2013) for read mapping and GATK (Van der Auwera et al., 2013) for variant detection. Three of the above-mentioned studies were interested in determining the concordance of SNP and genotype detection between RNA-seq and DNA-seq, the latter being the gold standard for SNP detection. However, these studies used only few samples (from 1 to 4) and had not at their disposal both RNA-seq and DNA-seq data on the same tissues of the same individuals.

In this context, this work aims at detecting SNPs from RNA-seq data in chicken. The first goal was to set up a procedure allowing SNP detection and genotype (GT) calling from RNA-seq data using reference tools (STAR for read mapping and GATK for SNP detection). We tested the SNP reliability according to three filters suggested by the GATK team and compared the detected SNPs with those obtained using DNA-seq data. This comparison was performed in two independent chicken populations for which RNA-seq and DNA-seq data were available on the same biological samples (i.e., the same tissue of the same individuals). In this paper, the workflow was used at the tissue level to provide results for RNA-seq experimental settings with only one analyzed tissue which represent a quite common case. This, however, corresponds to the least favorable case compared to multi-tissue experimental projects, since it does not allow cumulating the sequences from tissues per individual. We then analyzed the effects on the number of detected SNPs by this workflow performed at the tissue level when using additional tissues of a same population.

Because a large proportion of SNPs detected by RNA-seq was reliable, we further applied this procedure to 11 different chicken populations: a population derived from the wild Red Jungle Fowl population, an Egyptian Fayoumi population, six commercial and experimental laying hen populations and three commercial and experimental broiler populations. Our three goals were to (*i*) provide an estimation of the number of SNPs and GT that can be detected using RNA-seq data per tissue and population, (*ii*) present an overview of the predicted consequences of the SNPs located in coding regions, in particular, the number of high-confidence predicted loss-of-function variants, as defined in the work of gnomAD, and finally (*iii*) give an overview of the potential of RNA-seq for allele-specific expression (ASE) analysis by estimating the number of genes that could be analyzed for ASE with the number of SNPs detected per gene. We then identified the *cis*-regulated genes in the liver of 2 of the 11 populations using the phASER tool (Castel et al., 2016) and the proportion of cis-regulated hepatic genes shared by the two populations. Finally, we illustrated the possibility of using RNA-seq data to explore genetic diversity between populations using different hepatic RNA-seq SNP sets with variable percentage of severe predicted protein consequence.

## Materials and Methods

### RNA-Seq and DNA-Seq Data

Raw data of both DNA-seq and/or RNA-seq are available on the ENA and SRA archives under accession numbers: PRJEB28745 (RpRm DNA-seq and RNA-seq, Novo1 and Novo2, RNA-seq); PRJEB43829 (FLLL, DNA-seq); PRJNA330615 and PRJNA248570 (FLLL, RNA-seq); PRJEB26695 (red jungle fowl, RNA-seq); PRJEB34341 (Naked neck, RNA-seq); PRJEB34310 (Fayoumi, RNA-seq); PRJEB27455 (FrAg, RNA-seq); PRJEB43662 (Cobb, RNA-seq); PRJNA612882 (HerX, RNA-seq) (Fu et al., 2015). RNA sequencing was conducted on all samples using an Illumina HiSeq (Illumina, California, United States) system, with 2 × 150 bp or 100 bp. Libraries were prepared following Illumina’s instructions by purifying poly-A RNAs (TruSeq RNA Sample Prep Kit). Illumina adapters containing indexing tags were added for subsequent identification of samples.

For the comparison of SNPs detected by RNA-seq versus DNA-seq, we used two populations for which both data types were obtained from same liver samples collected on the same birds. The population A was composed of 15 birds from an experimental layer population (RpRm, PRJEB28745) composed of birds diverging for feed efficiency (Rp and Rm) after a 40-year diverging selection (Bordas et al., 1992). The population B was composed of 8 birds from an experimental broiler population (FLLL, PRJNA330615) composed of birds diverging for body fat content (FL and LL) (Roux et al., 2015).

For the rest of the work, we used RNA-seq data from 11 populations (see Additional File 1 for the detail of the number of birds, the tissues and the number of samples): a red jungle fowl population (called RJFh with 36 birds and 3 tissues); 3 broiler populations, the FLLL presented previously but here extended with 32 birds and 2 tissues) and two commercial ones, the Cobb 500 (Cobb Vantress, named Cobb with 48 birds and 2 tissues) and a 3-way cross produced by Heritage Breeders, LLC (named HerX, 23 birds and 1 tissue), 6 layer populations with 2 commercial brown-egg subpopulations from the Novogen company, Novo1 with 32 birds and 1 tissue and Novo2 with 40 birds and 2 tissues, 2 experimental brown-egg populations with the RpRm presented previously but here extended (with 88 birds and 5 tissues) and an experimental dwarf chicken layer line homozygous for the Naked Neck mutation (named LSnu with 16 birds and 2 tissues) and 2 other layer populations with a leghorn breed (FrAg) with 4 birds and 2 tissues) and the Fayoumi (FAyo), an Egyptian breed with 16 birds and 2 tissues; finally an experimental population (Rmx6) issued from crosses between 2 experimental lines (Frésard et al., 2014) with 19 embryos harvested from the same batch at embryonic day 4.5 (stage 26).

### RNA-Seq Read Mapping and Variant Detection

**FIGURE 2:**
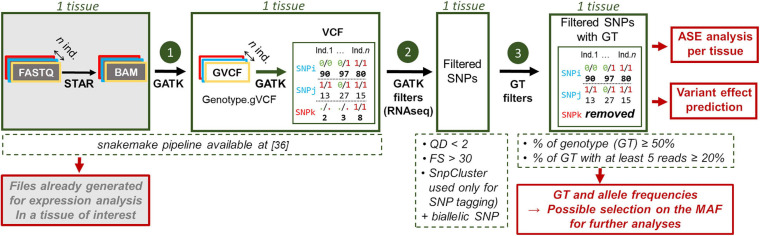
Workflow used to detect SNPs from RNA-seq data. The input files are indicated in gray. GATK filters: QD quality depth, FS: Fisher strand and SnpCluster: 3 or more SNPs in a sliding window of 35 bp. This last criterion was used only for tagging and not for filtering SNPs. For each SNP, are given the genotype (e.g., 0/0) for 3 individuals and under each genotype the associated read number (e.g., 90).

For all samples, RNA-seq variants were detected using the snakemake (Koster and Rahmann, 2012) pipeline, available at this reference: (GitLab, 2019). For each population, samples were analyzed by tissue. FASTQ files were trimmed for Illumina adapter using TrimGalore version 0.4.5 (Krueger, 2021). STAR v.2.5.2b (Dobin et al., 2013) was used with default parameters for the read mapping on the Gallus_gallus-5.0 reference genome, after the multi-sample 2-pass mapping procedure, with a GTF file enriched in long non-coding genes [available on http://www.fragencode.org (LNChickenAtlas); Section: Galgal5—Ensembl v94; Genome annotation: LNCextendedEns94.gtf.gz; (Jehl et al., 2020)]. Uniquely mapped reads (selected on a mapping quality score equal to 255) were then post-processed following the GATK best practices for RNA-seq data [duplicates were marked, reads overlapping intron were split and mapping quality score were reassigned, indel were realigned and base were recalibrated thanks to the known variants from Ensembl v94’s dbSNP (Ensembl, 2018)]. Variant detection was done for each sample using the “HaplotypeCaller” function of GATK (McKenna et al., 2010; DePristo et al., 2011; Van der Auwera et al., 2013) 3.7.0 with option “—stand_call_conf 20.0,” “—min_base_quality_score 10” and “—min_mapping_quality_score 20” (which are the defaults values), generating one gVCF file per sample. The “GenotypeGVCFs” function was then used with option “—stand_call_conf 20.0,” to jointly genotype all these samples into one VCF per tissue. The VCF file obtained at the end of the pipeline was then used as the input to two other steps, as summarized in Figure 2. First, biallelic SNPs were then extracted using the “SelectVariant” function with option “—selectType SNP—restrictAllelesTo BIALLELIC.” Variants were also filtered using “VariantFiltration” with two of the three suggested filters, “QD < 2” and “FS > 30,” as we discussed in the Results and Discussion section. Finally, we selected the SNPs with genotypes associated with each individual and that met the criteria established in results and Discussion section, i.e. (5.reads.DP) genotype CR ≥ 20% and CR ≥ 50%. Genotype and allele frequencies were then computed, making possible to work on SNPs selected on the minor allele frequency (MAF). These VCF files containing the SNP with their associated genotypes can be used for allele specific expression (ASE) analysis in each tissue of interest.

It is important to note that all previous treatments were conducted in this paper at the tissue level to provide SNP detection results for RNA-seq experimental settings with only one analyzed tissue, which is quite common and corresponds to the least favorable case. This implies that we had one bird’s genotype per tissue. For the multi-tissue analysis step of this paper, gVCF files generated per tissue were combined and genotypes were computed from all the tissues information using “CombineGVCFs” and “GenotypeGVCFs” generating per bird as many genotypes as tissues analyzed. Genotype concordance between tissues for a same bird was very high (∼99% of SNPs) and increased with coverage (see result section). Therefore, for the rare cases of discordance, we kept the genotype of the tissue with the highest coverage when they were different. However, outside from this study, for projects in which RNA-seq of different tissues per animal are available when the SNP detection analysis is started, we advise users of our pipeline to define in the first step a sample as a specific individual. This strategy allows to gain power in SNP detection by gathering all BAM tissue files per animal.

### DNA-Seq Read Mapping and Variant Detection

DNA-seq read mapping and variant detection were performed using standard tools. The BWA-MEM algorithm (Li, 2013) from BWA-0.7.17 was used with default parameters for the read mapping on the Gallus_gallus-5.0 reference genome (GCA_000002315.3). Variant detection was done for each sample using the “HaplotypeCaller” function of GATK (McKenna et al., 2010; DePristo et al., 2011; Van der Auwera et al., 2013) 3.7.0 with option “-variant_index_type LINEAR,” “-variant_index_parameter 128000,” “-mmq 30” and “-mbq 10 2,” generating one gVCF file per sample. The “CombineGVCFs” and “GenotypeGVCFs” (with “stand_call_conf 20.0” option) functions were then used to combine these gVCF into one VCF per population (one VCF for the 15 RpRm and one VCF for the 8 FLLL). Biallelic SNPs were then extracted using the “SelectVariant” function with option “—selectType SNP—restrictAllelesTo BIALLELIC.” Variant were filtered using “VariantFiltration” with all the recommended filters for DNA-seq: “FS > 60.0,” “QD < 2.0,” “SOR > 3.0,” “MQ < 40.0,” “MQRankSum < −12.5” and “ReadPosRankSum < −8.0.”

### Gene and Exon Expression Quantification

Gene expression was quantified with RSEM (Li and Dewey, 2011) v.1.3.0, at the gene-level, using the GTF file LNCextendedEns94.gtf.gz available on http://www.fragencode.org (LNChickenAtlas; section Galgal5) and corresponding to the genes from the Ensembl annotation used as reference, extended with lncRNAs loci available in other public databases (NCBI, NON-CODE, etc.) (Muret et al., 2017). To compute expression at the exon level, we used FeatureCount v1.6.2 (Liao et al., 2014) with options -t “exon” and -g “exon_id.” We defined for each exon a metric called RpKb (Read per Kilobase) as the mean number of reads mapped at the exon divided by its length in kilobases. To define an expression threshold, we compared the expression of exons to the expression of a set of randomly selected loci in the genome as done previously in Jehl et al. (2020). The background noise corresponds to the expression of a set of artificial loci randomly distributed across chicken chromosomes 1–33 using the “shuffle” function from the BEDTools suite v2.29 (Quinlan and Hall, 2010). These artificial loci had the same length distribution as the LNC genes known to be the less expressed compared to PCG and were positioned at a distance of at least 5kb of the closest known transcribed regions. The expression of these randomly selected regions was well below the expression of the exons. We set as an expression threshold for the exons a log_1__0_(RpKb + 1) value of 0.5, corresponding to the first quartile of expression in both RpRm and FLLL (see Additional File 2).

### Variant Functional Predictions

Variant Effect Predictor (VEP) v92 (McLaren et al., 2016) with the GTF file enriched in long non-coding genes (“—gtf”) was used for effect prediction of 9,496,283 SNPs. “—everything” and “—total_length” options were applied to respectively, obtain SIFT score predictions and length of cDNA, CDS and proten positions (Ng, 2003; Sim et al., 2012).

### Detection of Homopolymers and Exon-Exon Junctions

Regions with 5 or more repeated nucleotides (homopolymers) and regions spanning 5 bp of each extremity of a junction were detected using home-made scripts.

### Hierarchical Clustering Analysis

The hierarchical clustering was performed on a set of 67,341 SNPs obtained using liver RNA-seq data from the 10 populations presented in Table 1 (liver unavailable for Rmx6). This set corresponds to the SNPs common to the 10 populations and passes the GT criteria (see “Results and discussion”) for each population. The analysis was produced by using the function “snpgdsHCluster” of the R (R Core Team, 2019) package SNPRelate v1.8.0 (Zheng et al., 2012).

**TABLE 1:** SNP counts per population retained at each step of the selection.

Population				Total SNP			Selected GT					Selected GT and MAF ≥ 10%				
			
Pop.	#ind.	#smpl.	#tiss.	Liver^a^	Multi-tiss.^b^	b/a	Liver^c^	Multi-tiss.^d^	d/c	c/a	d/b	Liver^e^	Multi-tiss.^f^	f/e	e/a	f/b
RJFh	36	72	3	1,050,035	2,604,288	2.48	265,750	578,726	2.18	0.25	0.22	152,029	319,268	2.10	0.14	0.12
Cobb	48	96	2	3,771,992	5,464,266	1.45	949,127	1,678,364	1.77	0.25	0.31	558,020	952,445	1.71	0.15	0.17
FLLL	32	64	2	1,729,800	2,033,207	1.18	535,228	1,109,324	2.07	0.31	0.55	368,280	714,523	1.94	0.21	0.35
HerX	23	23	1	1,332,709	1,332,709	1.00	481,314	481,314	1.00	0.36	0.36	307,859	307,859	1.00	0.23	0.23
Novo1	32	32	1	1,459,352	1,459,352	1.00	447,594	447,594	1.00	0.31	0.31	264,804	264,804	1.00	0.18	0.18
Novo2	44	104	2	1,289,199	2,146,975	1.67	390,195	738,109	1.89	0.30	0.34	243,892	449,446	1.84	0.19	0.21
RpRm	112	286	5	1,841,778	4,032,988	2.19	555,928	1,279,458	2.30	0.30	0.32	307,049	631,868	2.06	0.17	0.16
Rmx6	19	19	1	–	2,123,217	–	–	715,822	–	–	0.34	–	483,379	–	–	0.23
FrAg	4	7	2	1,247,253	1,732,440	1.39	784,397	1,055,772	1.35	0.63	0.61	520,277	583,742	1.12	0.42	0.34
Lsnu	16	32	2	1,487,176	2,284,902	1.54	590,399	836,800	1.42	0.40	0.37	384,720	534,938	1.39	0.26	0.23
Fayo	16	32	2	1,320,244	2,033,207	1.54	496,412	698,932	1.41	0.38	0.34	288,464	396,446	1.37	0.22	0.19
Mean				1,652,954	2,477,050	1.54	549,634	874,565	1.64	0.35	0.37	339,539	512,611	1.55	0.22	0.22
Union	382	767		5,490,587	9,496,283		1,685,406	3,276,615				1,255,554	2,243,766			
Intersection				221,374	241,960		67,341	73,223				2,442	1,442			

*In columns—Pop., population; #ind., bird number; #smpl., sample number; #tiss., tissue number; Multi-tiss., Multi-tissues. Superscripts are used to show which ratio are presented. Total SNP: SNPs detected at the population level (i.e., with at least one ALT allele); Selected GT: SNPs with at least 50% of genotypes (CR ≥ 50%) and 20% of GT with reads ≥ 5 reads [(5.reads.DP)genotypeCR ≥ 20%, see “Results and discussion”]; Selected GT with minor allele frequency (MAF) ≥ 10%.*

*In lines—Union: SNPs detected in at least one population; Intersection: SNPs detected in each of the 10 populations (i.e., each population has at least one ALT allele).*

### Allele-Specific Expression (ASE) Analysis

Prior to the quantification of allele specific expression, sequences need to be aligned against masked version of the genome to avoid favoring reference alleles. At the population level, polymorphic (allele frequency < 100%) and bi-allelic filtered (GATK—FS and QD criteria) SNP were extracted using the GATK “SelectVariants” tool. These last variants were then used to mask the reference genome using “maskfasta” tool from the BEDTools suite v2.29. Tissue sample sequence were aligned to this masked version of the genome using the multi-sample 2-pass mapping procedure of STAR 2.6. Non-duplicated (“MarkDuplicates” function from GATK 4.1.2, with “READ_NAME_REGEX” set to null) properly paired (if paired sequences) uniquely mapped reads (samtools 1.9 with –f 2 and –q 255 options) were selected. “SplitNCigar” tool from GATK were finally used to split alignment overlapping exon/intron junction and rescaled mapping quality. The phASER tool (Castel et al., 2016) and its downstream tool phASER Gene AE were used to detect ASE among the liver samples of the RpRm and FLLL populations. Briefly, phASER phases, in each sample, SNPs from a user-provided VCF, using the reads from the previously processed BAM file of the sample. This produces a list of haplotypes upon which phASER counts the number of reads associated to each “super-allele.” Then, in each sample, phASER Gene AE selects one haplotype per gene, using the genes’ boundaries from a user-provided BED file, allowing the study of the gene’s ASE using the selected haplotype.

Using base quality of 10, and mapping quality of 20, we provided a VCF containing the SNP that met the criteria established here. After selection of one haplotype per gene using phASER Gene AE, we considered only the genes represented by a haplotype with at least 10 reads associated to at least 1 super-allele. To assess ASE in each sample, we screened for read number imbalance between the super-alleles using a binomial test (*binom.test* R function) with the null hypothesis that, for a given gene, each super-allele had the same number of associated reads. *P*-values were corrected using the Benjamini-Hochberg method (Benjamini and Hochberg, 1995) with a false discovery rate of 0.05. We considered a gene to be ASE if it presented a significant read number imbalance in at least 2 samples.

## Results and Discussion

### SNP Detection by RNA-Seq: Genome Location

**FIGURE 3:**
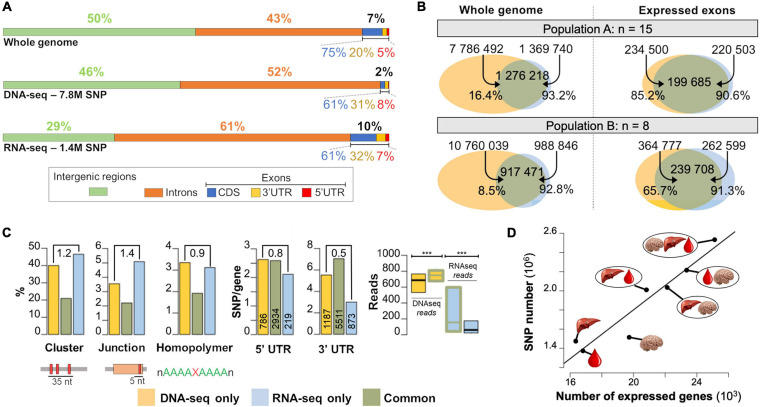
Differences and common features of SNPs detected by RNA-seq and DNA-seq. **(A)** Percentage of the genome comprising each type of feature (top) and the proportion of SNPs detected by DNA-seq (middle) and RNA-seq (bottom) across these genomic features. **(B)** Number of SNPs detected by DNA-seq only (yellow set), RNA-seq only (blue set) and by both methods (gray set) at the whole genome level (left) and expressed exon level (right) in two independent populations A (*n* = 15, layers) and B (*n* = 8, broilers). **(C)** Left: Percentages of SNPs in SNP clusters (i.e., 3 or more SNPs in a sliding window of 35 bp, as per GATK definition), in junctions, homopolymers, in population A by DNA-seq only, RNA-seq only and both methods (common). Middle: Number of SNPs detected in 5′ and 3′UTR by gene (*Y*-axis) and the gene number (vertical numbers) in population A. The ratio “RNA-seq specific SNPs/DNA-seq specific SNPs” is indicated at the top of each plot. Right: read supporting SNP distribution at the population level in DNA-seq or RNA-seq data. **(D)** Evolution of the number of detected SNPs as a function of the number of expressed genes using one tissue alone or groups of tissues. Tissues used were liver (figured as a liver), blood (figured as a blood drop) and hypothalamus (figured as a brain).

We compared the repartition of the SNPs detected by DNA-seq and RNA-seq among different genomic regions (Figure 3A). The chicken genome is composed at equal parts of intergenic (50%) and genic (50%) sequences, with 43% of introns and 7% of exons. As expected, DNA-seq SNPs were mostly distributed across the non-coding part of the genome (46% in intergenic regions, 52% in introns) and at a lower proportion (2%) in exonic regions. This distribution is expected since coding regions are generally under stronger selection pressure than non-coding regions (Zhao et al., 2003). With RNA-seq (all the samples being systematically treated with DNAse), we expected to find most of the SNPs in exonic regions, which represent the majority of expressed regions. However, the majority of the detected SNPs were located in intronic (61%) and intergenic (29%) regions. Higher SNP counts in intronic regions can be explained by the presence of unspliced transcripts (premature transcripts), very lowly expressed compared to spliced transcripts, but sufficiently to be supported by reads, and by the lower selection pressure on these regions compared to the exons. SNPs located in “intergenic regions” are likely to be located in new genes or in not yet annotated part of genes (particularly 3′UTR and 5′UTR). Within exons, the proportion of SNPs in 3′UTR, 5′UTR and CDS were similar between RNA-seq and DNA-seq (32, 7, 61%), but significantly different from the proportion of these regions in the genome (20, 5, 75%) showing a lower selection pressure in 3′UTR regions than in CDS regions.

### SNP Detection by RNA-Seq: Concordance With Those Detected by DNA-Seq

We detected SNPs using either RNA-seq or DNA-seq data obtained from the liver of the same 15 laying hens (see population A in Figure 3B, left). We found 7,786,492 biallelic SNPs using the DNA-seq data filtered with the standard criteria of GATK (see section “Materials and Methods”) and considered them as reliable. Using the RNA-seq data filtered with some of the filters suggested by GATK (see section “Materials and Methods” and comments below), we found 1,369,740 SNPs. As expected, the number of SNPs detected with RNA-seq is much lower than that in DNA-seq, because only variants present in transcribed regions were detected. Note that the impact of all these filters on the SNP number was provided in the Additional File 3 for DNA-seq and RNA-seq and was quite low, more than 98% of SNP were kept after filtering whatever the population.

To provide a meaningful comparison of both methods, we used the SNPs detected in expressed exons, assessed using RNA-seq with the metric described in section “Materials and Methods.” We detected in population A 147,474 expressed exons among the 162,145 exons of the 16,814 expressed genes (on average 8.8 exons per gene). As shown in Figure 3B right, in these exons, 85.2% of the 234,500 SNPs detected by DNA-seq were also detected by RNA-seq. In population B, which was composed of only 8 broiler chickens, we found that 65.7% of the SNPs detected with DNA-seq in the expressed exons were also detected by RNA-seq. Assuming SNPs detected by DNA-seq represent the “truth,” these percentages represent the sensitivity, or recall, of RNA-seq for SNP detection. This difference in RNA-seq sensitivity between populations A and B is likely due to the number of samples per population (15 versus 8), that affects the extent to which reads at each position are accumulated across the samples (see Figure 1).

Concerning the precision of RNA-seq, among the 220,503 SNPs detected by RNA-seq in population A, and the 262,599 SNPs from population B, 90.6 and 91.3%, respectively, were detected by DNA-seq 20X showing a reasonable precision of RNA-seq for the SNP detection. These results are consistent with the findings of Guo et al. (2017), who compared the percentage of SNPs detected using RNA-seq versus exome sequencing and found around 85% concordance. Regarding the 9.4% (20,818 SNPs) RNA-seq specific SNPs, we analyzed different factors that could underlie their detection to highlight those that should be treated with caution (Figure 3C) and verify these factors in DNA-seq variants set or in the set of variants called by both methods. We consider the SNPs detected by DNA-seq as true since DNA-seq are now routinely used for SNP detection with the well-proven GATK filters. First, we observed that a large proportion of RNA-seq specific SNPs (46.6%) and DNA-seq specific SNP (40.0%) belonged to a “SNP cluster” (i.e., 3 or more SNPs in a sliding window of 35 bp, as per GATK definition) (Figure 3C). This filter is one of the three filters proposed by GATK for RNA-seq SNP detection, but not for DNA-seq detection and the GATK team notes that these filters are not definitive and should be validated by users. Therefore, in the light of these observations, we decided not to remove the “SNP clusters” from our RNA-seq dataset as for DNA-seq dataset, but only to flag them as belonging to a so-called SNP cluster. Indeed, this filter removed 39,783 true SNPs (i.e., True positives detected by both DNA-seq and RNA-seq methods) and consequently the benefit of the precision increase (from 90.6 to 93.5) by removing “SNP clusters” was too small relatively to the recall decrease (from 0.85 to 0.68). The 20,818 RNA-seq specific SNPs can be explained by other factors of lowest impact: (*i*) 5.09% were located at 5 bp or less of an exon-exon junction, *versus* 3.55% for those detected only by DNA-seq; the corresponding ratio, that is significantly greater than 1 (1.4, *p* ≤ 10^–17^, χ^2^ test), was expected since RNA-seq deals with spliced transcripts (Figure 3C) and therefore RNA-seq read mapping by the aligner is more complicated and more error-prone than DNA-seq read mapping. Since most of them are also observed in DNA-seq, we consider that the SNPs in the vicinity (i.e., 5 bp) of the junctions can be kept, but should be validated by another technique. Note that these SNPs represent only 0.48% of the total SNPs detected by RNA-seq. (*ii*) 3.1% were located in low complexity regions, defined as repetition of at least 5 identical nucleotides, versus 3.4% for the ones detected only by DNA-seq (Figure 3C). (*iii*) 2.7 and 5.5 SNPs per gene for RNA-specific SNPs were observed in 5′UTR and 3′UTR regions, respectively, with a fewer 3′UTR SNPs compared to those detected by DNA-seq only (0.5, *p* ≤ 10^–16^, χ^2^ test) (Figure 3C). This may be due to the fact that mature transcripts undergo exonucleases action, degrading their 3′ extremities and causing their absence in RNA-seq libraries (Gallego Romero et al., 2014). (*iv*) Last, another factor that could be responsible for these RNA-seq specific SNPs is RNA editing, however, according to the literature, it is unlikely that most of the remaining SNPs are due to this mechanism. In mammals, in which RNA editing is well studied, Adenosine-to-Inosine (A-to-I) editing due to ADAR1 and ADAR2 enzymes is the most common editing form and mostly occur in inverted pairs of Alu interspersed repeats (Porath et al., 2014). In chicken *Alu*-like family of interspersed repeats also exist and they are called CR1 (Olofsson and Bernardi, 1983). These editing events tend to occur in clusters, a phenomenon called hyper-editing that introduces ≥ 20 mismatches in the sequencing reads (Carmi et al., 2011), that are therefore discarded by the aligner either because of a multi-mapping or no mapping. The prevalence of editing is still discussed: RNA editing is rarely detected when standard mapping filters are used, as shown in mice (Lagarrigue et al., 2013a) and chickens (Frésard et al., 2015; Roux et al., 2016; Shafiei et al., 2019), with less than 200 events, and in humans (Kleinman et al., 2012; Tan et al., 2017) with less than 1000 events per tissue. By contrast, RNA editing is frequently detected when working in repeated regions and rescuing unaligned reads (Picardi et al., 2017). Finally, we observed that SNPs detected only by one method were supported by significantly less reads (either of RNA- or DNA-seq) than the SNPs detected by both methods (Figure 3C).

### SNP Detection by RNA-Seq: Impact of the Number of Tissues That Are Analyzed

Using blood and hypothalamus samples collected on the same 15 animals (population A), we studied the effect of detecting the SNPs in more than one tissue. RNA-seq from each tissue was not generated at the same time and have been analyzed separately at different occasions. Results are displayed in Figure 3D. We detected 1,369,740 SNPs in the liver (as previously stated), 1,481,627 in the blood and 1,511,909 in the hypothalamus, while 16,814 genes were expressed in liver, 16,346 in blood, and 19,733 in hypothalamus. As expected, using combinations of two or three tissues, the number of detected SNPs increased in relation with the number of expressed genes (spearman correlation = 0.96, *p* = 3 × 10^–3^) by cumulating the information on all tissues in which a same gene is more or less expressed. Note that here, we have used our pipeline in a sub-optimal manner, by analysing RNA-seq data per tissue instead of combining the tissues together to increase detection power and reliability. For projects in which RNA-seq from different tissues per animal are all available before SNP detection analysis, we advise users to pool for each animal the RNA-seq files. For SNPs detected in more than one tissue, the concordance between genotypes detected in different tissues was very high, 98.9% without read filtering. Considering genotypes supported by at least 5 reads (respectively 10 reads) the concordance raised to 99.5% (respectively 99.9%).

### Genotype (GT) Calling by RNA-Seq Importance of Genotype Call Rate (CR) and Read Depth at the Individual Scale for Selecting SNPs With Enough Reliable Genotypes for *in fine* Calculating Genotype and Allele Frequencies

While reliable SNPs can be detected in the population thanks to some individuals that bear them, it does not necessarily mean that there are enough reads for each individual to produce a genotype (GT). This was exemplified in Figure 1B by the brown cells (SNPs 3 and 4), for individuals 4 and 5 (“stress” group) for SNP 3 or most of the individuals of the population for SNP 4. These cases are quite frequent in practice because of gene expression variability between individuals in a given tissue, especially when different conditions are analyzed or also when a SNP is located in an intron of an immature transcript (weakly abundant compared to the mature transcript). Therefore, genotype call rate (CR), defined as the percentage of individuals with a genotype in the population, can be highly variable (e.g., from 16 to 100% in Figure 1B, right) from one SNP to another, depending on the number of reads observed in each individual (DP per individual). With 20X DNA-seq data, most of the SNP have a genotype CR close to 100%, as depicted in Figure 1A.

**FIGURE 4:**
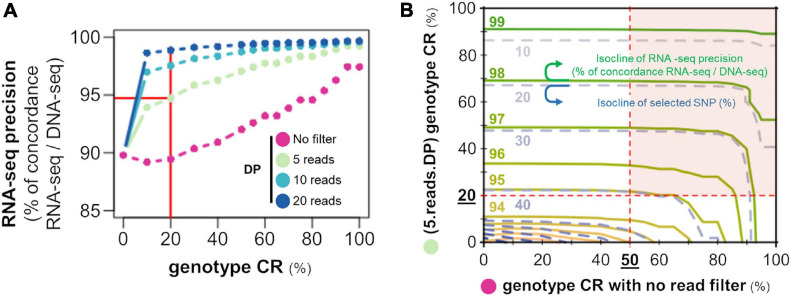
The passage from SNP to GT necessitates a read depth threshold. **(A)** Evolution of the percentage of genotype concordance between RNA-seq and DNA-seq (*y*-axis) for the 15 RpRm birds as a function of genotype call rate in the population (CR: *x*-axis) supported by at least 5 (light green), 10 (light blue), or 20 (dark blue) reads or without read filter (purple curve). The red lines correspond to the criteria used in the further analysis (CR ≥ 20% with a DP ≥ 5 reads) and the corresponding RNA-seq precision. **(B)** Isoclines of the percentage of genotype concordance between RNA-seq and DNA-seq (solid green lines) and of the percentage of SNPs selected out to the original set (dashed blue lines) according to the CR with no read filter (*x*-axis) and the CR with at least 5 reads [(5.reads.DP) genotype CR (%), *y*-axis]. Red surface: SNPs selected after filtering on (5.reads.DP) genotype CR ≥ 20% and a CR ≥ 50%.

These observations indicate that a genotype can be observed with a certain call-rate but its reliability will depend on the DP supporting it. The GT reliability was estimated by the genotype concordance between RNA-seq and DNA-seq, assuming that GT detected by DNA-seq represents the truth. This concordance corresponds to the precision of RNA-seq for GT calling. We tested the RNA-seq precision according to different criteria. First, we conjointly studied in Figure 4A the effects of the criteria “genotype CR” and “DP supporting the genotype” on the RNA-seq precision (genotype concordance between RNA-seq and DNA-seq). We found a concordance (of roughly 90%) when no threshold was applied on the DP (purple line); it increased to around 95% for a CR ≥ 20% with a DP ≥ 5 reads and over 97% for a CR ≥ 20% with a DP ≥ 10 reads. We then evaluated the impact of the CR alone (without a DP threshold, *x*-axis) versus the CR with a DP ≥ 5 reads (*y*-axis), on the genotype concordance between RNA-seq and DNA-seq (solid green isoclines) and on the number of SNPs selected according to the different criteria (dashed blue isoclines) (Figure 4B).

Interestingly, only the CR with DP ≥ 5 reads have an effect on the genotype concordance and the percentage of selected SNPs, while no such effect is observed for the no DP filtering CR (*x*-axis) comprised between 0 and 50%, as shown by the horizontal isoclines. Hence, we propose for our subsequent analysis on different RNA-seq datasets to select SNPs within the red surface of Figure 4B with a (5.reads.DP) genotype CR ≥ 20% ensuring a concordance (precision) of almost 95% and a CR ≥ 50% ensuring a sufficient number of GT per SNP to calculate the allelic frequencies. We can note that most of the SNPs on this surface have a genotype concordance of more than 97%. We can also note in most of the populations analyzed in the next section that more than 98% of SNPs with (5.reads.DP) genotype CR ≥ 20% have a CR ≥ 50% (Additional File 4).

### Number of SNPs and Genotypes Detected by RNA-Seq in 11 Populations

As shown in Table 1 which gives an overview of the SNP diversity in 11 chicken populations, we detected between 1.1 and 3.8 M SNPs per population using liver RNA-seq datasets. Using all the tissues available (1–5 tissues depending on the population), we detected more SNPs, consistently with our previous result (see Figure 3D): between 1.7 and 5.5 M SNPs with a fold increase of × 1.18 to × 2.48 depending on the number and nature of analyzed tissues. Across populations and using all tissues, we found a total of 9.5 M SNPs having at least one alternative allele in at least one population (SNP union), and 241,960 SNPs that had at least one alternative allele in each of the 11 populations (SNP intersection). The union of our SNPs contains 23% (2,175,528) yet-unreported SNPs in the reference Ensembl v94 dbSNP database [(Ensembl, 2018): 21 M SNPs]. The intersection of our SNPs contains 5.1% (12,203 SNPs) of the SNPs present in the 600K genotyping array (Kranis et al., 2013).

We then filtered SNPs on genotype call rate and read depth (Table 1, “Selected GT”) and found between around 0.4 and 1.7 M SNPs using all tissues, 37% of the SNPs observed previously. These results on 11 populations show that a large number of SNPs (two thirds) were detected at the population level thanks to the accumulation of reads across all individuals of the population, but that within each individual, read counts are not sufficient to reliably determine a genotype. Nevertheless, the number of SNPs with a genotype per population remains in the order of magnitude of several hundred thousand to a few millions with a union of 3.3 M and an intersection of 73,223 SNPs. In the liver, for which data was available in all but one population (Rmx6), the union and intersection are of the same order of magnitude: 1.7 M and 67,341 SNPs, respectively. After selecting for a MAF (minor allele frequency) ≥ 10% in order to discard rare SNPs or those resulting from sequencing errors, the number of SNPs was halved in all populations with a grand total of 2.2 and 1.3 M for the multi-tissue and liver union, respectively. As expected, the intersection drastically decreased to approximatively 2,000 SNPs, since this set corresponds to the SNPs with a MAF ≥ 10% in each of the 11 populations. The list of the 9.5 M of SNPs including 3.3 M with a GT and 2.2 M with MAF ≥ 10% is available on http://www.fragencode.org/lnchickenatlas.html.

### Rare Deleterious Variants Detection in the Populations

**FIGURE 5:**
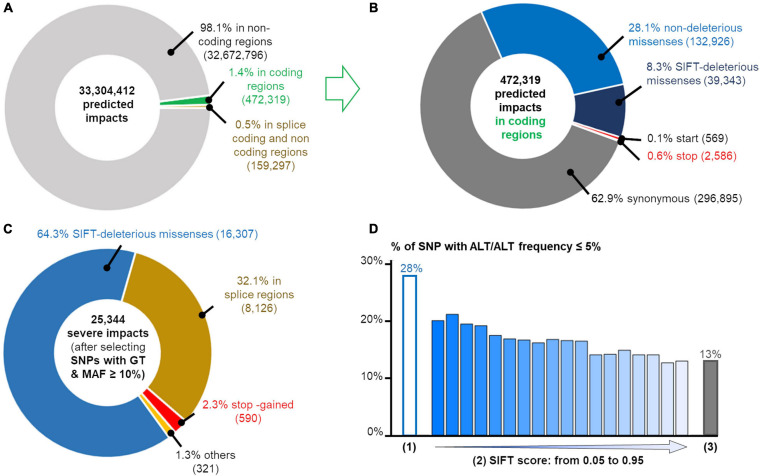
Annotation of 9,496,283 SNPs using Variant effect Predictor (VEP) (McLaren et al., 2016). **(A)** Distribution of variant effect predictions among non-coding (light gray), splice regions related to coding and non-coding genes (orange) and coding (green) regions. **(B)** SNP annotation in coding regions: synonymous (dark gray), non-deleterious (light blue) and deleterious (blue) missenses, and consequences affecting stop (red) and start (orange) codons. Total number of consequences are indicated between parentheses. **(C)** Annotation of SNPs predicted as deleterious and filtered according to the GT criteria (as defined previously) and a MAF ≥ 10% in at least one population. **(D)** Percentage of SNP with a very low frequency (≤ 5%) of ALT/ALT genotype for three SNP sets: (1) = the 25,344 deleterious SNP described on the left; (2) = the tolerated SIFT-missense SNP according to the SIFT score and (3) = the synonymous SNP set. The splice sites correspond to the donor or acceptor splice sites of coding and long non-coding genes.

We predicted the impacts of the 9,496,283 SNPs detected in at least one population using the VEP tool (McLaren et al., 2016) which predicts the potential consequences of the SNPs in each of the transcripts carrying them: we found 33,304,412 consequences. As expected, the vast majority of the SNPs affected non-coding regions (Figure 5A) and among the 472,319 SNPs affecting a coding-region, a majority were synonymous (63%) or non-deleterious missense (28%) as shown in Figure 5B.

**FIGURE 6:**
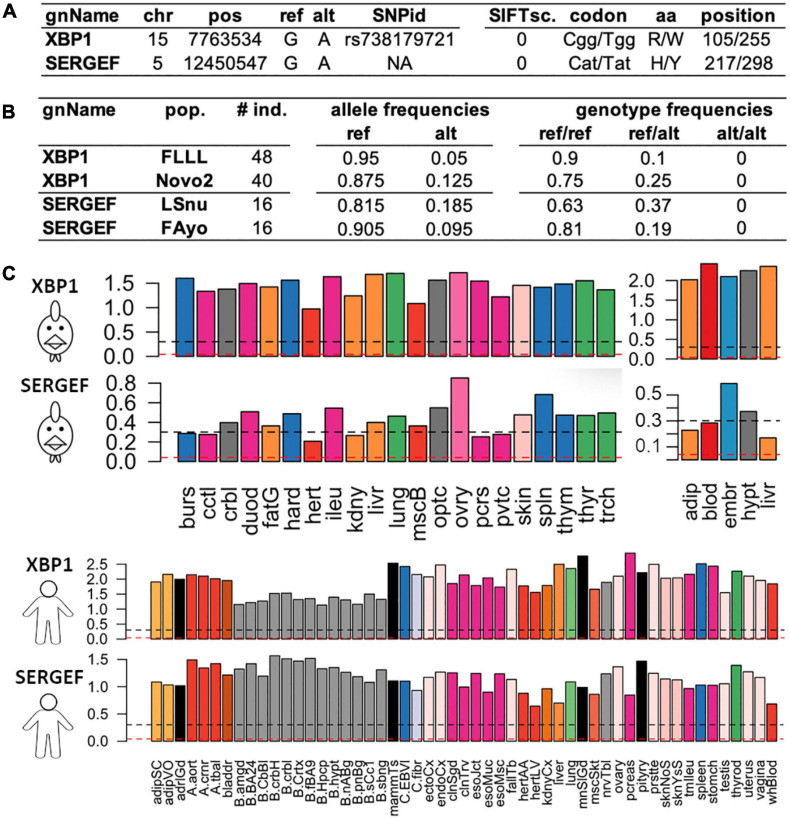
Two examples of deleterious missense SNPs impacting two protein coding genes (XBP1 and SERGEF). **(A)** genomic position of the SNP with its identifier (SNPid) in Ensembl dbSNP and its impact on the protein with SIFTsc.: SIFT score, codon/modified codon, amino acid/modified amino acid and its position in the protein. **(B)** pop.: population with the individual size (# ind.) observed per population and the frequencies of the alleles and genotypes. **(C)** Tissue expressions [log10(TPM + 1)] in chicken using two datasets composed of 21 tissues (ERP014416) (left) and 5 tissues RpRm population) (right) and in human through the 53 tissues from the GTEX consortium (The GTEx Consortium, 2020). Abbreviations for the 21-tissue dataset: burs, bursa of Fabricius; cctl, cecal tonsils; crbl, cerebellum; duod, duodenum; fatG adipose tissue around the gizzard; hard, harderial gland; hert, heart; ileu, ileum; kdny, kidney; livr, liver; lung, lung; mscB breast muscle; optc, optical lobe; ovry, ovary; pcrs, pancreas; pvtc, proventriculus; skin, skin; spln, spleen; thym, thymus; thyr, thyroid gland; trch, trachea; and for the 5-tissue dataset: adip, abdominal adipose tissue; blod, blood; embr, 4.5 day embryos; hypt, hypothalamus; livr, liver; for more details in these 3 datasets and associated samples see Jehl et al. (2020). Black dashed line: gene expression with TPM ≥ 1 and red dashed line: TPM ≥ 0.1.

Among all these predictions, we focused on the predicted consequences with the most severe putative impacts as defined by the gnomAD consortium, which only considers the PCG (Protein Coding Genes) (Genome Aggregation Database Consortium et al., 2020): variants in the splice regions, start and stop codon loss or stop codon gain even if the severity of the latter depends on its position in the coding sequence. We also added missense variants with a SIFT score ≤ 0.05. As reported by gnomAD (The GTEx Consortium, 2020), these SIFT-deleterious SNPs generally have a low frequency in the populations and can be mistaken for sequencing errors. Hence, it is crucial to select SNPs with genotypes (as defined previously) and a MAF ≥ 10% in at least one population (i.e., the ALT allele observed for example at least 4 times in a population of 16 individuals as for FAyo and LSnu populations) to make sure that the deleterious allele is not spurious. Thanks to our data from 382 individuals from the 11 populations, we listed a total of 25,344 strong predicted impacts (Figure 5C), corresponding to 14,496 SNPs and 67,58 genes, among them were 590 predictions of stop gained (404 genes), 8,126 of a coding or non-coding gene splice site change (donor and acceptor), 16,307 SIFT-predicted deleterious missenses and 321 other predictions (start lost, stop lost). Out of these 25,344 deleterious-predicted impacts, we found 5,654 (22%) predictions corresponding to 2,872 (20% of 14,496 SNPs) variants in 1,884 genes for which the homozygous ALT/ALT genotype was absent, in all populations in which the ALT allele was detected and, respectively, 7,740 (31%) predictions corresponding to 4,072 (28% of 14,496 SNPs) variants in 2,515 genes with ALT/ALT frequency ≤ 5%. The analysis of tolerated missense SNP show that the higher the SIFT score (i.e., tolerated variant), the lower the percentage of SNP with a low frequency (≤ 5%) of ALT/ALT genotype (Figure 5D). The same analysis performed with 217,119 synonymous variants showed lower percentages with 9% SNPs with ALT/ALT genotype absent and 13% SNP with ALT/ALT frequency ≤ 5%. Such results are compatible with a homozygous state which is lethal or strongly negatively selected (28 versus 13%, *p* ≤ 10^–20^, χ^2^ test), suggesting an important role for the genes associated to these variants with severe-predicted impact. Such variants obtained using RNA-seq data constitute a new complementary resource to Ensembl dbSNP allowing to explore variants (deleterious or not) according to their genotypic and allelic frequencies in different populations of a farm species. For example, two deleterious missense SNPs (SIFT-score = 0) are presented in Figure 6. One is already reported in dbSNP (Ensembl genome browser 94, 2020) and affects XBP1 protein by changing a positive charged amino acid (Arginine, R) into an aromatic and hydrophobic amino acid (Tryptophan, W) (Figure 6A). This SNP is observed in two of the ten analyzed populations, FLLL and Novo2, with 5 and 10 heterozygous birds among 48 and 40 animals analyzed, respectively, whereas no ALT/ALT homozygous birds were observed (Figure 6B). This gene is ubiquitously expressed in chicken as in human (Figure 6C). It codes the “Tax-Responsive Element-Binding Protein 5” transcription factor which has important cellular and physiological roles related to the “unfolded protein response” pathway in the endoplasm reticulum [(Lee et al., 2003) and for review (Glimcher et al., 2020)] and also to hepatic insulin resistance (Zhou et al., 2011).

The second SNP, not reported in dbSNP, affects the SERGEF protein (alias DelGEF) by changing an aromatic, hydrophobic and positive charged amino acid (Histidine, H) into an unchanged amino acid (Tyrosine, Y) (Figure 6A). This SNPs was observed in two populations, LSnu and Fayoumi, with 6 and 3 heterozygous birds among 16 animals, respectively, whereas no ALT/ALT homozygous birds were observed. This gene is also relatively ubiquitously expressed in chicken as in human (Figure 6C). The functions of this gene, which codes the “Secretion Regulating Guanine Nucleotide Exchange Factor” seem to be poorly known: 9 publications found in PubMed with the key words, SERGEF or DELGEF. As illustrated by these two examples (XBP1 and SERGEF), the analysis of various populations allowed to increase the number of rare deleterious variants detected.

### Potential for Allele-Specific Expression Analysis in Various Populations

**FIGURE 7:**
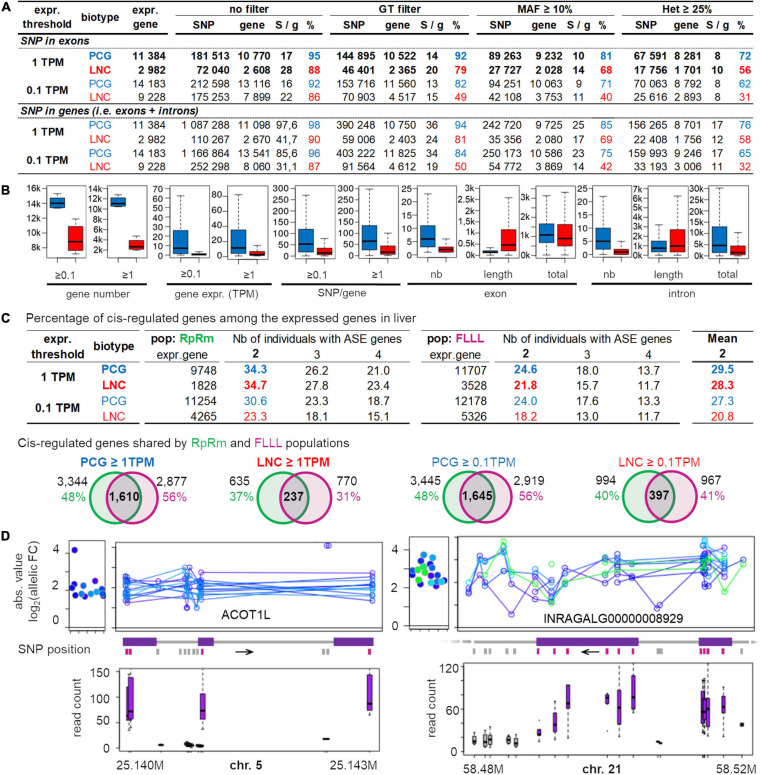
Overview of the analyzable genes for allele-specific expression in the liver of various populations for two gene biotypes, Protein Coding Gene (PCG) and Long Non-Coding gene (lncRNA), at two gene expression thresholds (0.1 TPM and 1 TPM) and for 3 filters. **(A)** Average numbers for all populations analyzed here. These average numbers are provided for both PCG (blue) and lncRNA (red) biotype, with minimum expression of 0.1 or 1 TPM (“expr. threshold”), and considering only the SNPs in exons (top part) or in the whole gene, i.e., in both exons and introns (bottom part). **(B)** Feature of lncRNA and PCG. **(C)** Percentage of gene with a significant allele specific expression in two populations RpRm (in left) and FLLL (in right) in comparison to the expressed gene number. Venn diagrams provide the number of ASE genes (in at least 2 individuals) shared by RpRm and FLLL populations. **(D)** Overview of the ASE of ACOT1L (left) and INRAGALG00000008929 (right). For each ASE sample, absolute values of the log2 allelic fold-change are represented at the gene-level (left of the panels) and for each SNP located in the haplotype used by phASER (right). Boxplot of the read number associated to each SNP are represented (bottom), in purple for the SNP located in exons and in gray for those in introns. FC, fold-change; chr, chromosome.

Allele-specific expression (ASE) analysis requires a heterozygous SNP in the expressed feature, to test an eventual imbalance in the expression between the two parental chromosomes. Usually, the expression is evaluated using RNA-seq and the SNPs are detected using DNA-seq, which is expensive when working on a dozen or more individuals. Since we have shown that RNA-seq allows detecting a large number of reliable SNPs in expressed regions, we studied in this section, the potential of RNA-seq data for performing ASE analysis. To this end, the Figure 7A provides the average numbers of genes across various populations, having at least one SNP with different filters (SNPs with an associated GT, a MAF ≥ 10% and an heterozygous status in at least 25% of the population). We also indicated the average SNP number per gene (column “S/g”) to give an idea of the RNA-seq potential to test ASE along the gene. We indicated the results for two types of genes: the protein-coding genes (PCG) and the long non-coding genes (lncRNA), which are increasingly considered as important regulators of gene expression but are also known to be less expressed than PCG (Derrien et al., 2012; Muret et al., 2017; Le Béguec et al., 2018). This is the reason why we studied two expression thresholds: 0.1 and 1 TPM commonly used when working on lncRNA and PCG, respectively. Finally, results in Figure 7A are presented either for SNPs detected in exons (i.e., mature transcripts) (top) or for SNPs detected in exons or introns hence including immature transcripts (bottom).

The first key result is that the number of genes with at least one SNP are similar in both cases (exons only versus exons + introns), meaning that there are enough SNPs to study ASE in exonic regions only, i.e., mature transcript, despite a much lower number of SNPs per gene when SNPs are only selected in exons (Figures 7A,B). When working with exonic SNPs, there are on average 17–28 SNPs without filter (8–10 SNPs after all filters) per gene showing the possibility to test ASE along genes. Despite a lower exonic length in lncRNA compared to the PCG (Figure 7B), this number is higher for lncRNA compared to PCG (22–28 versus 15–17) probably due to lower selective pressure on lncRNA compared to PCG. The second key result, after applying 2 filters (GT and MAF ≥ 10%), is that 81% of PCG (9,232) and 68% of lncRNA (2,028) expressed at TPM ≥ 1 are analyzable for ASE. These numbers decreased a little after applying an additional filter related to the heterozygosity percentage, with 72% of PCG and 56% of lncRNA (i.e., about 10,000 genes). The variability of this “ASE analyzable genes” percentage is moderate (Additional File 5): on average 72% from 65 to 89% with an except for the “RpRm” (48%) probably due to its high consanguinity and its large size, the filter of 25% of heterozygosity impacting more the populations with a larger sample size. The same tendencies regarding the percentage of genes that can be analyzed were observed for the PCG (TPM ≥ 0.1) and for lncRNA (both for TPM ≥ 0.1 and ≥ 1) (Additional File 5). We can note that the selected lncRNA percentage satisfying the filters is always lower than the selected PCG percentage (−15% for genes with an expression ≥ 1TPM and −30% for genes with an expression ≥ 0.1TPM). This is mainly due to the lower expression of lncRNA compared to PCG (Jehl et al., 2020; Figure 7B), despite higher sequence variability for the former.

### *Cis-*Regulated Genes in the Liver of Two Populations

To provide an estimation of the number of *cis-*regulated genes in one tissue, we performed an ASE analysis of the liver samples of the RpRm and the FLLL populations using phASER and its downstream tool, phASER Gene AE, that phase SNPs at the gene level (see also section “Materials and Methods”). Using exonic and intronic SNPs and selecting genes having one haplotype with at least 10 reads, we found for genes with an hepatic expression ≥ 1 TPM, that in average 29% of the expressed PCG or lncRNA genes were *cis-*regulated (∼34% for RpRm and ∼23% for FLLL) (Figure 7C). For lncRNA with hepatic expression ≥ 0.1 TPM which represents most of this biotype, we found a lower percentage of *cis-*regulated genes (21%) because they are less expressed and some of them did not have more than 10 reads for at least one “super-allele” analyzed by phASER (see section “Materials and Methods”). Interestingly, among these *cis-*regulated genes, ∼50% and 37% are shared by both populations for the protein-coding genes and long non-coding genes, respectively (Figure 7C). Two examples of *cis-*regulated genes are provided in Figure 7D with a PCG, ACOT1L (ENSGALG00000008752), and a lncRNA, INRAGALG000000089295. Overall, these numbers are consistent with the literature: Zhuo et al. (2017) found that 15% of the genes were *cis-*regulated in chicken embryo liver, and Lagarrigue et al. (2013b) found a similar number in mice liver. In humans, the GTEx consortium (The GTEx Consortium, 2020) found that 26% (4,415) of the expressed genes (17,243) were *cis*-regulated in the liver.

### Diversity Exploration Using RNA-Seq Variants

Finally, we explored genetic links between populations using the genotypic frequencies of SNPs detected by RNA-seq, which represent a set of SNPs, which may be under a larger selective pressure than those used in genotyping SNP chips. Indeed, the latter are considered as having a neutral effect, while most the SNPs present in our data are located in expressed regions and affect proteins to some extent (from almost neutral synonymous to deleterious stop gained).

**FIGURE 8:**
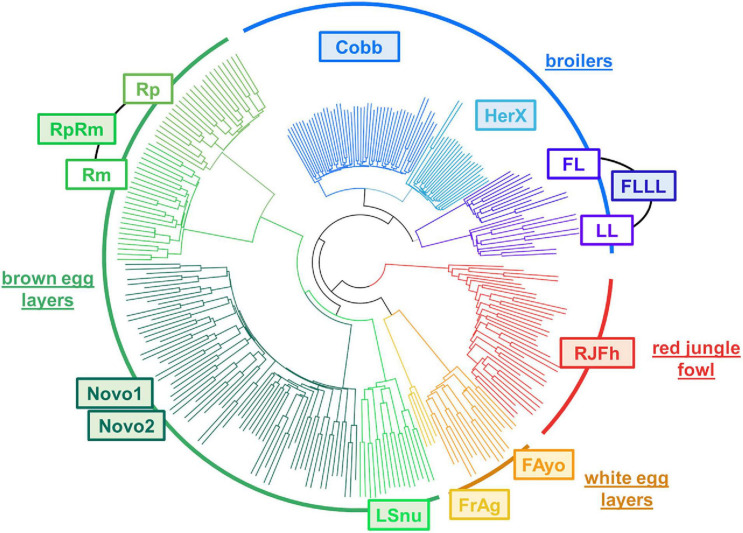
Hierarchical clustering of 10 chicken populations using the 67,341 SNP intersection set with GT obtained using liver RNA-seq data. The hierarchical clustering was performed using the “snpgdsHCluster” from the package SNPRelate v1.8.0 (see also section “Materials and Methods”).

The classification in Figure 8 was produced using the intersection of SNPs with GT of the 10 populations with a liver presented in Table 1 (67,341 SNP set). This classification is consistent with the known chicken population history, indicating that these SNPs detected by RNA-seq and their associated genotypes allow distinguishing different populations. The classification separated clearly the RJFh (red circle arc with a Red Jungle Fowl population, used here to represent the “ancestral” population), then the broilers (blue circle arc), the brown-egg layers (dark green circle arc), and the cream- or white-egg layers (brown circle arc with Fayoumi breed and Fr-Ag population which is an experimental leghorn line). We also observed the expected sub-groups within these 3 types of populations: the commercial lines (Novo1 and Novo2 for the layers, Cobb and HerX for the broilers) separated from the experimental lines (RpRm for the brown-egg layers, FLLL for the broilers). Interestingly for these 2 last populations, this SNP set shows a clear distinction between two subpopulations that have been divergently selected for a specific trait: Rp and Rm divergent for the residual feed intake and FL and LL divergent for body fat whereas the two Novogen populations (Novo1 and Novo2) are not distinct. We can note that the SNPs predicted as “missense” by VEP and “deleterious” by SIFT provide the same classification between the populations as the one shown in Figure 8 (data not shown).

## Conclusion

We show here that RNA-seq data, which are cheaper to generate and store compared to DNA-seq data, can be a reliable resource for performing different analyses based on polymorphism detection. By comparing DNA-seq and RNA–seq results generated from the same animals in two independent chicken populations, this study provides a workflow to produce reliable SNPs and genotypes from RNA–seq data. We ran through this pipeline 767 RNA–seq of 382 birds from 11 populations and provided a per-population estimation of the average genotyped SNPs count per tissue (more than 550,000) and an overview of the predicted consequences of SNPs located in coding regions. In particular, thanks to this large RNA-seq dataset, we identified 440 genes containing a stop-gained impact, known to be rare because of their potentially severe impact, especially when located in the first third of the coding sequences (133 genes). In a companion study (Degalez et al., submitted), we checked the possible existence of more than one SNP in a given codon, that could “rescue” a stop-gained situation. We then gave an overview across 11 populations of genes that could be analyzed for ASE, i.e., having at least one SNP allowing to distinguish expression from both chromosomes. We applied phASER on liver RNA-seq data of two populations and identified around 21 to 30% of *cis*-regulated genes depending on the analyzed population and the gene biotype (PCG versus lncRNA), these results were consistent with other studies conducted in other species.

This study represents a first step to more ambitious projects that could analyze tens of thousands of available RNA-seq datasets to build a GTEx-like atlas reporting *cis*- and *trans*- genetic associations with gene expression, as previously performed in human (The GTEx Consortium, 2020) and more recently in cattle (Liu et al., 2020).

## Data Availability Statement

The datasets presented in this study can be found in online repositories. The names of the repository/repositories and accession number(s) can be found in the article/Supplementary Material.

## Ethics Statement

Ethical review and approval was not required for the animal study because all data have been obtained from public databases as described in the materials and methods, section “DNA-Seq and RNA-Seq Data.”

## Author Contributions

CK and SLa conceived the study and coordinated the study. FJ, LL, CD, BA, MT-B, BB, TB, DG, HA, SF, SD, EG, FP, TZ, and SLa participated to the set-up of the experimental design and sample collection. LL, CD, and SLe carried out all RNA extractions. OB generated RNA-seq libraries and sequencing. FJ, FD, MB, CK, and SLa performed bioinformatic processing of the RNA-seq data and carried out the whole analyses. FL, PB, and MC participated to bioinformatic analyses. FJ, FD, and SLa drafted the manuscript. MB, FL, BA, MT-B, BB, HA, SF, SD, EG, TZ, FP, and CK helped to improve the manuscript. All authors read and approved the final version.

## Conflict of Interest

The authors declare that the research was conducted in the absence of any commercial or financial relationships that could be construed as a potential conflict of interest.

## Supplementary Material

Additional File 1 Detail per population of the number of birds, tissues and samples.

Additional File 2 Boxplot of the exon expression in log10 (RpKb+1) for both RpRm and FLLL populations compared to noise. Noise corresponds to the expression of a set of randomly selected loci in the genome.

Additional File 3 Effect of GATK filters on SNP numbers with RNA-seq and DNA-seq data. For RNA-seq, the analyses were performed in different tissues and populations. For DNA-seq, the analyses were performed for the two populations, RpRm with 15 individuals and FLLL with 8 individuals. Abbreviations for the tissue: adip: abdominal adipose tissue, blod: blood, embr: embryos, hypt: hypothalamus, livr: liver, spln: spleen. “rawSNP”: SNP detected before the filters’ application. “filtered biallelic SNP”: SNP detected after the filters’ application.

Additional File 4 SNP counts per population with each genotype filters independently.

Additional File 5 Overview of the analyzable genes for allele-specific expression in the liver of various populations for Protein Coding Gene (PCG) and Long Non-Coding gene (lncRNA), at two gene expression thresholds (0.1 TPM and 1 TPM) with the mean across the populations (“mean”) and the coefficient variation (“CV”), i.e., standard deviation divided by mean. “expr. gene”: expressed genes, “gene“: genes bearing the SNP, “S/g”: number of SNPs divided by the number of gene, “%“: proportion of genes bearing the SNPs relative to the expressed genes. The 4 filters: no filter, GT satisfying our criteria (“GT filter”), with MAF ≥ 10% (“MAF ≥ 10%”) and heterozygosity rate ≥ 25% (“Het. ≥ 25%”).
