# Supplementary material for: RNA-Seq Data for Reliable SNP Detection and Genotype Calling: Interest for Coding Variant Characterization and Cis-Regulation Analysis by Allele-Specific Expression in Livestock Species
Source: Front Genet. 2021 Jun 28;12:655707. doi: 10.3389/fgene.2021.655707 (PMC8273700; doi:10.3389/fgene.2021.655707)
Supplement: Additional File 2 — Boxplot of the exon expression in log10 (RpKb+1) for both RpRm and FLLL populations compared to noise. Noise corresponds to the expression of a set of randomly selected loci in the genome. [file Table_2.DOCX]

**
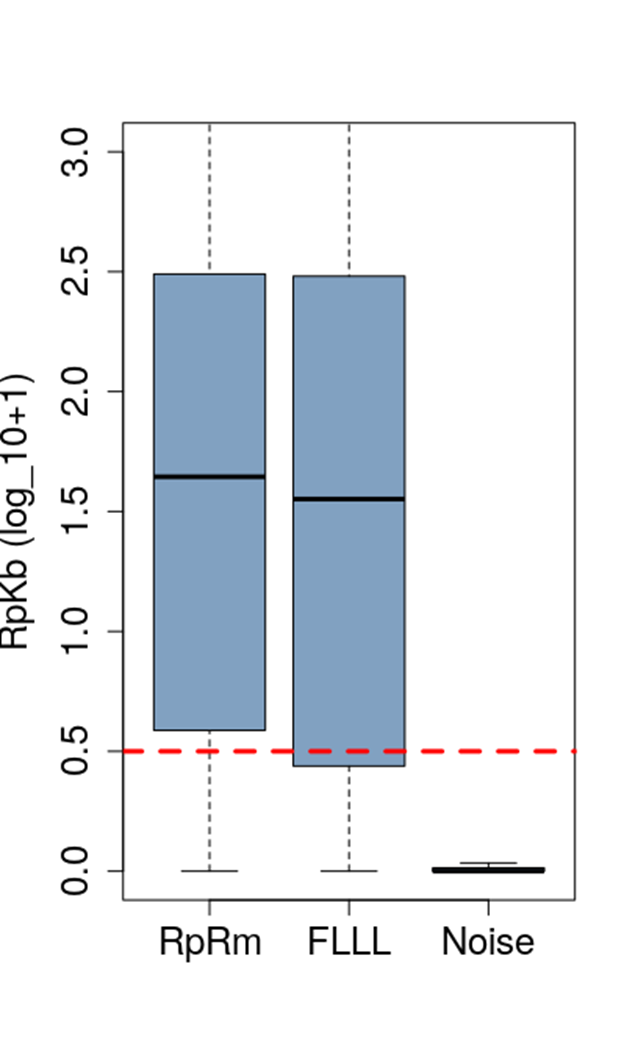
**

**Additional File 2: Boxplot of the exon expression in log10(RpKb+1) for both RpRm and FLLL populations compared to noise.**

Noise is representing by the expression of a set of randomly selected loci in the genome
